# Polydatin alleviates bleomycin‐induced pulmonary fibrosis and alters the gut microbiota in a mouse model

**DOI:** 10.1111/jcmm.17937

**Published:** 2023-09-04

**Authors:** Jia Yang, Xiawei Shi, Rundi Gao, Liming Fan, Ruilin Chen, Yu Cao, Tingzhen Xu, Junchao Yang

**Affiliations:** ^1^ Department of Respiratory and Critical Care Medicine The First Affiliated Hospital of Zhejiang Chinese Medical University Hangzhou China; ^2^ The First Clinical College Zhejiang Chinese Medical University Hangzhou China

**Keywords:** bleomycin (BLM), gut microbiota, lung injury, polydatin, pulmonary fibrosis

## Abstract

To investigate the effect and mechanism of polydatin on bleomycin (BLM)‐induced pulmonary fibrosis in a mouse model. The lung fibrosis model was induced by BLM. The contents of TNF‐α, LPS, IL‐6 and IL‐1β in lung tissue, intestine and serum were detected by ELISA. Gut microbiota diversity was detected by 16S rDNA sequencing; R language was used to analyse species composition, α‐diversity, β‐diversity, species differences and marker species. Mice were fed drinking water mixed with four antibiotics (ampicillin, neomycin, metronidazole, vancomycin; antibiotics, ABx) to build a mouse model of ABx‐induced bacterial depletion; and faecal microbiota from different groups were transplanted into BLM‐treated or untreated ABx mice. The histopathological changes and collagen I and α‐SMA expression were determined. Polydatin effectively reduced the degree of fibrosis in a BLM‐induced pulmonary fibrosis mouse model; BLM and/or polydatin affected the abundance of the dominant gut microbiota in mice. Moreover, faecal microbiota transplantation (FMT) from polydatin‐treated BLM mice effectively alleviated lung fibrosis in BLM‐treated ABx mice compared with FMT from BLM mice. Polydatin can reduce fibrosis and inflammation in a BLM‐induced mouse pulmonary fibrosis model. The alteration of gut microbiota by polydatin may be involved in the therapeutic effect.

## 1 INTRODUCTION

Idiopathic pulmonary fibrosis (IPF) is a chronic, progressive and fibrotic disorder characterized by the replacement of healthy lung tissue with an abnormal extracellular matrix (ECM), the excessive deposition of ECM proteins, and the disruption of alveolar architecture, resulting in decreased lung compliance, disrupted gas exchange and ultimately respiratory failure and death.^1^ IPF is the most common form of idiopathic interstitial pneumonia, whose incidence has risen over time. It is estimated that the prevalence rate of IPF varies from 2.8 to 18 per 100,000 people per year in Europe and North America.^2^ IPF is more common in men and is rare in younger people under 50 years (median age at diagnosis is approximately 65 years)^3^, ^4^; despite the variable and unpredictable disease course, the median survival time after diagnosis is 2–4 years.^1^

The microbiota is mainly composed of bacteria, fungi and viruses, among which the most widely and deeply studied composition is the bacterial symbiotic flora.^5^ Studies have concluded that gut microbiota and respiratory diseases have been closely related in recent years. Many chronic lung diseases (such as asthma and chronic obstructive pulmonary disease (COPD)) are usually associated with chronic gastrointestinal diseases (e.g. inflammatory bowel disease or irritable bowel syndrome).^6^ Up to 50% of adults with inflammatory bowel disease and 33% with irritable bowel syndrome have clinical manifestations of pulmonary involvement with no history of acute or chronic respiratory diseases.^7^ There are, however, very few studies exploring how the gut microbiota contributes to IPF.

Polydatin (C_20_ H_22_ O_8_) is a naturally active single crystal compound extracted from the roots and stems of Polydanum.^8^ Polydatin is the glycoside form of resveratrol, which has been shown to have a wide range of therapeutic effects, such as anti‐inflammatory,^9^ antitumor,^10^ nerve regeneration‐promoting^11^ and mitochondrial function‐improving^12^ effects, in various diseases. Some resveratrol is metabolized by the gut microbiota, while studies suggest that the therapeutic potential of resveratrol may be related to changes in the beneficial microbiota due to its interaction with the gut microbiota.^13^ Polydatin has pharmacological effects similar to those of resveratrol in some ways but still has unique specificity. To our knowledge, no study has revealed the interaction between polydatin and gut microbiota.

This study aimed to examine the role of polydatin in IPF and explore its mechanism. Additionally, the modification of gut microbiota and the function of faecal microbiota transplantation (FMT) were determined.

## 2 MATERIALS AND METHODS

### 2.1 Animals

Male C57BL/6 mice (8–10 weeks of age) provided by SJA Laboratory Animal Co., Ltd, Shanghai, China. All mice were housed in specific pathogen‐free conditions in an animal facility with controlled humidity (50%–60%) and temperature (22–24°C) and under a 12 h light–dark cycle (7 AM to 7 PM) with free access to food and drink. All procedures were sanctioned by the ethics committee of Zhejiang Chinese Medical University.

### 2.2 Modelling and treatments

The pulmonary fibrosis model was established by intratracheal BLM instillation in mice. Briefly, the mice were anaesthetised by intraperitoneal injection of pentobarbital (50 mg/kg). Anaesthetised mice were placed in a supine position and fixed on a sterile operating table, with the head high and the feet low and the neck skin fully exposed. Ophthalmic scissors were used to cut the neck skin approximately 2 cm along the midline after routine disinfection of the anterior neck area. The trachea of mice was fully exposed after blunt dissection, the trachea was punctured by bending the 1 mL syringe needle at an obtuse angle, the needle was inserted approximately 1.5 cm along the wall of the trachea, and the prepared BLM (5 mg/kg [5000 IU/kg] according to the body weight of mice^14^) or the equivalent amount of saline was dripped. After administration, the syringe was withdrawn, and the mice were placed vertically on the fixed plate and rotated along the long axis for approximately 2 min to distribute the BLM evenly into the alveolar cavity as previously described.^14^ Local skin disinfection was performed to prevent infection after suturing the skin.

A total of 24 mice were equally divided into four groups (*n* = 6) with different tracheal instillation solutions and different follow‐up treatments: (1) Control group: intratracheal administration and intragastric administration of normal saline; (2) BLM group: intratracheal administration of BLM as above mentioned and intragastric administration of normal saline; (3) Control + polydatin group: intratracheal administration of normal saline and intragastric administration of polydatin (40 mg/kg,^15^, ^16^ once per day); (4) BLM + polydatin group: intratracheal administration of BLM and intragastric administration of polydatin (40 mg/kg, once per day). Intragastric administration began on the second day of intratracheal administration by oral gavage needles. Four weeks later, the mice were sacrificed and samples were collected.

A mouse model of antibiotic (Abx)‐induced bacterial depletion was developed. Mice to be used for faecal transplantation were treated with Abx in advance. Mice were fed with drinking water mixed with four antibiotics (ampicillin, neomycin, metronidazole and vancomycin) for 3 weeks following the method described previously.^17^

### 2.3 Sample collection

The blood sample was collected, and the supernatant (serum) was stored at −80°C for further experiments. The faeces were collected for 16S rDNA sequencing or faecal transplantation. The left lung tissues and/or intestinal tissues were collected for pathological and ELISA analyses. The right lung was collected, and incubated at 60°C for 3–4 days to remove all moisture. Subsequently, the dry weight was measured, and the ratio of wet‐to‐dry weight was calculated.

### 2.4 Preparation of faecal microbiota suspension and transplant

Fresh faeces were collected from the control, BLM, control + polydatin and BLM + polydatin groups. Fresh faeces were collected from mice in the morning using the stress defecation method. The lower abdomen of the mice was gently pressed, and four to five complete faeces were collected from each mouse and stored in a sterilized centrifuge tube. The faeces of all mice in the target group were placed in the same centrifuge tube. The collected fresh faeces were added to sterilized saline at a ratio of 100 mg:1 mL, mixed by vigorous vortexing and centrifuged at 4°C for 3 min at 1200 rpm. Then, 200 μL supernatant was taken for enema administration in Abx‐treated mice (once per day for 2 weeks). The faecal microbiota supernatant from the control group, BLM group, control + polydatin group, and BLM + polydatin group were named control FMT, BLM‐FMT control‐Poly‐FMT, and BLM‐Poly‐FMT, respectively. The Abx‐treated mice were divided into four groups, and each group was named according to the control or BLM model and the origin of faecal microbiota to be transplanted: (1) Control + Control‐FMT; (2) BLM + BLM‐FMT; (3) Control + Control‐Poly‐FMT; and (4) BLM + BLM‐Poly‐FMT.

### 2.5 Sequencing analysis of gut microbial diversity (16S rDNA sequencing)

QIAamp Fast DNA Stool Mini Kit (Qiagen, Germany) was used for DNA extraction of faecal microbiota. Universal primers were designed using the gene fragment of the conserved region V3V4 of bacterial 16S rDNA, and PCR amplification was performed using the forward primer 341F: CTACGGGNGGCWGCAG and the reverse primer 805R: GACTACHVGGGTWTCTAATCC. The sequence of the V3V4 region was sequenced on the Illumina NovaSeq system.

### 2.6 α‐ and β‐diversity index

α‐diversity is an indicator of reaction richness, diversity and evenness. α‐diversity was evaluated using QIIME2 software. The observed species, ACE, and Chao1 indices were used in this study. β diversity refers to the replacement rate of species along the environmental gradient among different communities or the dissimilarity of species composition. β‐diversity was evaluated using principal coordinate analysis (PCoA) and nonmetric multidimensional scaling (NMDS). The above analysis was completed using the R language (4.02) and QIIME2 (QIME2‐2020.2) as previously described.^18^

### 2.7 Histopathological analysis

The mice were euthanized under anaesthesia. For the haematoxylin and eosin staining, the lung tissues were quickly removed after opening the thoracic cavity, washed using precooled PBS solution, fixed in 10% neutral formalin, routinely embedded in paraffin and sectioned 48 h later. The paraffin sections were dewaxed with xylene, rehydrated with gradient concentrations of alcohol, washed with PBS and immersed in haematoxylin staining for 10 min. Stained slices were observed under an inverted microscope. For the Masson staining, paraffin‐embedded sections were deparaffinized with xylene, dehydrated with a gradient of alcohol, washed in PBS, and stained for 10 min with a 1:1 mixed Weigert's haematoxylin solution. After being differentiated for 5–15 s with acidic alcohol differentiation solution, the sections were coloured for 3 min with Masson staining solution and stained for 5–10 min with Ponceau S staining solution. The stained slices were observed under an inverted microscope.

### 2.8 Immunohistochemical expression of collagen I and α‐SMA

The sections were routinely deparaffinized and rehydrated in gradient concentrations of alcohol. Antibody incubation was performed after antigen retrieval, blocking of endogenous peroxidase and blocking of nonspecific binding sites. The primary antibodies used were: collagen I (14695‐1‐AP, Proteintech, Wuhan, China) and α‐SMA (14395‐1‐AP, Proteintech). After overnight incubation, the subsequent steps were completed using a Pv 9000 General Purpose Two‐Step Immunohistochemical Detection Kit and DAB chromogenic solution.

### 2.9 Western blot analysis

Cells were lysed using RIPA lysis buffer (Beyotime, Shanghai, China) to obtain protein samples. After measuring the protein concentration with the BCA Kit (Beyotime), the corresponding volume of protein was added to the loading buffer (Beyotime), mixed well and heated in a boiling water bath for 5 min to denature the protein. Electrophoresis was carried out to separate the proteins. The membrane transfer was carried out on an ice bath with a current of 220 mA for 120 min. The membranes were rinsed for 1–2 min, put into the blocking solution for 60 min at room temperature, and incubated with primary antibodies against collagen I (14695‐1‐AP, Proteintech), α‐SMA (14395‐1‐AP, Proteintech) or GAPDH (60004‐1‐IG, Proteintech) overnight at 4°C. The membranes were washed and incubated with the secondary antibody (horseradish peroxidase‐labelled goat anti‐rabbit IgG, 1:5000, Beijing Kangwei Century Biotechnology Co., Ltd., Beijing, China) for 1 h at room temperature. After dripping the developer solution onto the membrane, signal detection was performed using a chemiluminescence imaging system (Bio‐Rad).

### 2.10 ELISA

TNF‐α, lipopolysaccharides (LPS), IL‐6, and IL‐1β in the lung tissue, intestine and serum of mice were detected in each group according to the instructions of the ELISA kit (RUXIN Biotech, Quanzhou, China). All operations were carried out in strict accordance with the instructions.

### 2.11 Statistical analysis

The experimental data obtained are expressed as the mean ± SD. GraphPad Prism 8.0 software was used for the *t*‐test or one‐way anova, and the Tukey method was used for multiple comparisons. When *p* < 0.05, the difference was considered statistically significant.

## 3 RESULTS

### 3.1 PF modelling and functional investigation of polydatin

**FIGURE 1:**
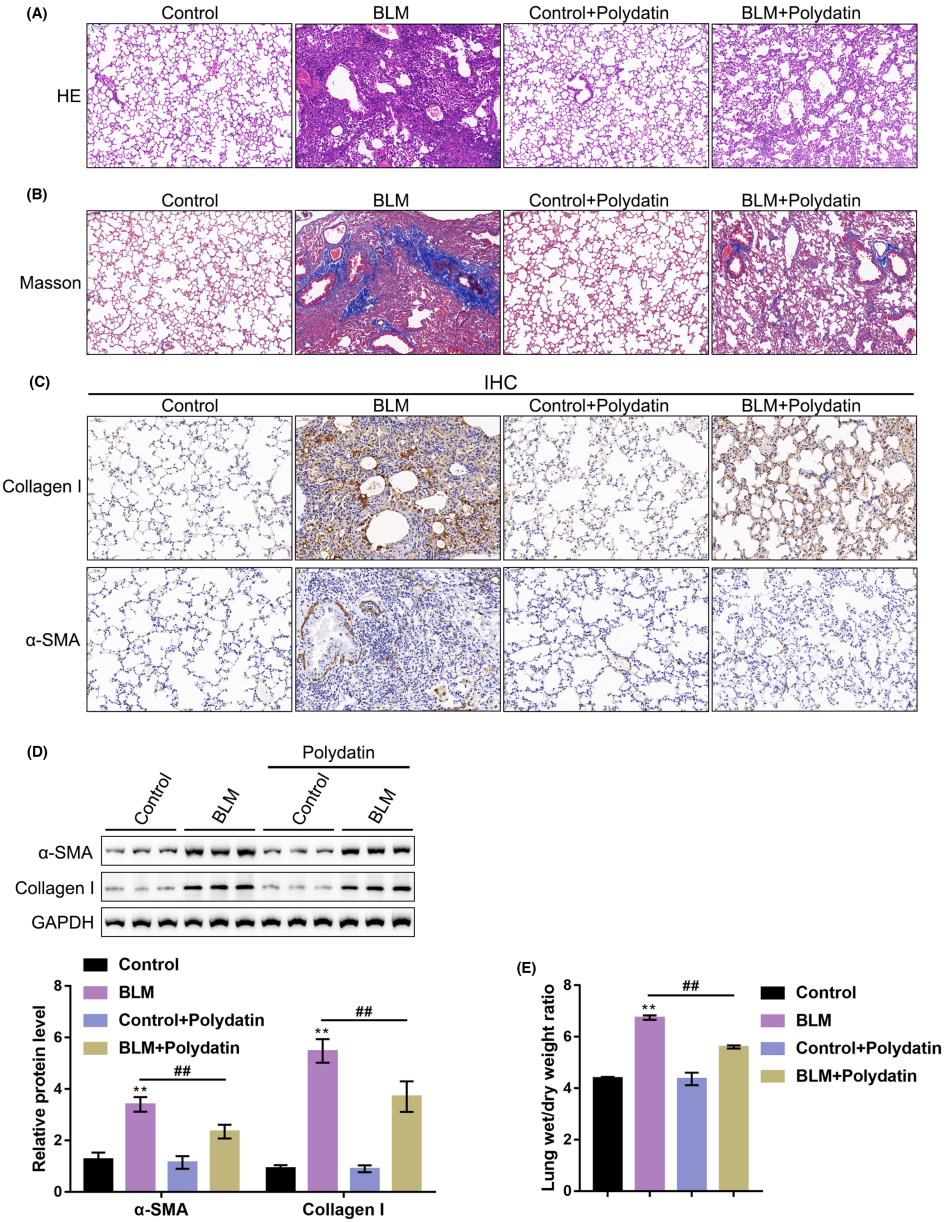
Pulmonary fibrosis modelling and functional investigation of polydatin. (A) Haematoxylin and eosin staining was performed to detect the pathological changes in lung tissue in each group; (B) Masson staining was performed to detect collagen deposition in the lung tissue of mice; (C) The expression levels of collagen I and α‐SMA in the lung tissue of mice were detected by IHC; (D) Western blotting was performed to detect the expression levels of collagen I and α‐SMA; (E) The W/D ratio of lung tissue in each group. **p < 0.01, compared to the control group; *##p <* 0.01, compared to the BLM group.

The pathological changes and collagen deposition in mouse lung tissues were evaluated using haematoxylin and eosin and Masson staining. Figure 1A shows that the alveolar structure was clear in the control group, the alveolar septa were normal and no inflammatory cell infiltration was observed. Similar trends were observed in the control + polydatin group, in which no alveolar pathological structure was found. In the BLM group, the alveolar septum was thickened, inflammatory cells were infiltrated, and fibroblast hyperproliferation and fibrotic areas were observed. Compared with the BLM group, fibrotic foci were significantly reduced in the BLM + polydatin group (Figure 1B).

Masson staining showed consistent results, as no collagen deposition was found in the lung tissue of the mice in the control group and the control + polydatin group, while collagen deposition was the most severe in the BLM group. Compared with the BLM group, collagen deposition was significantly reduced in the BLM + PD group. These results indicated that the BLM model was successfully established and that polydatin could effectively ameliorate BLM‐induced lung injury and collagen deposition in the mouse model. The levels of fibrotic markers, collagen I and α‐SMA, were also measured by IHC and Western blot. As shown in Figure 1C,D the levels of collagen I and α‐SMA were significantly increased in the BLM model, and polydatin significantly reduced the levels of these two markers. Furthermore, the W/D ratio of the BLM model was significantly increased, whereas polydatin treatment partially reduced the W/D ratio (Figure 1E). These findings indicated that the BLM model was successfully established and that polydatin could partially ameliorate BLM‐induced PF.

### 3.2 Effects of polydatin on inflammatory factors in the BLM model

**FIGURE 2:**
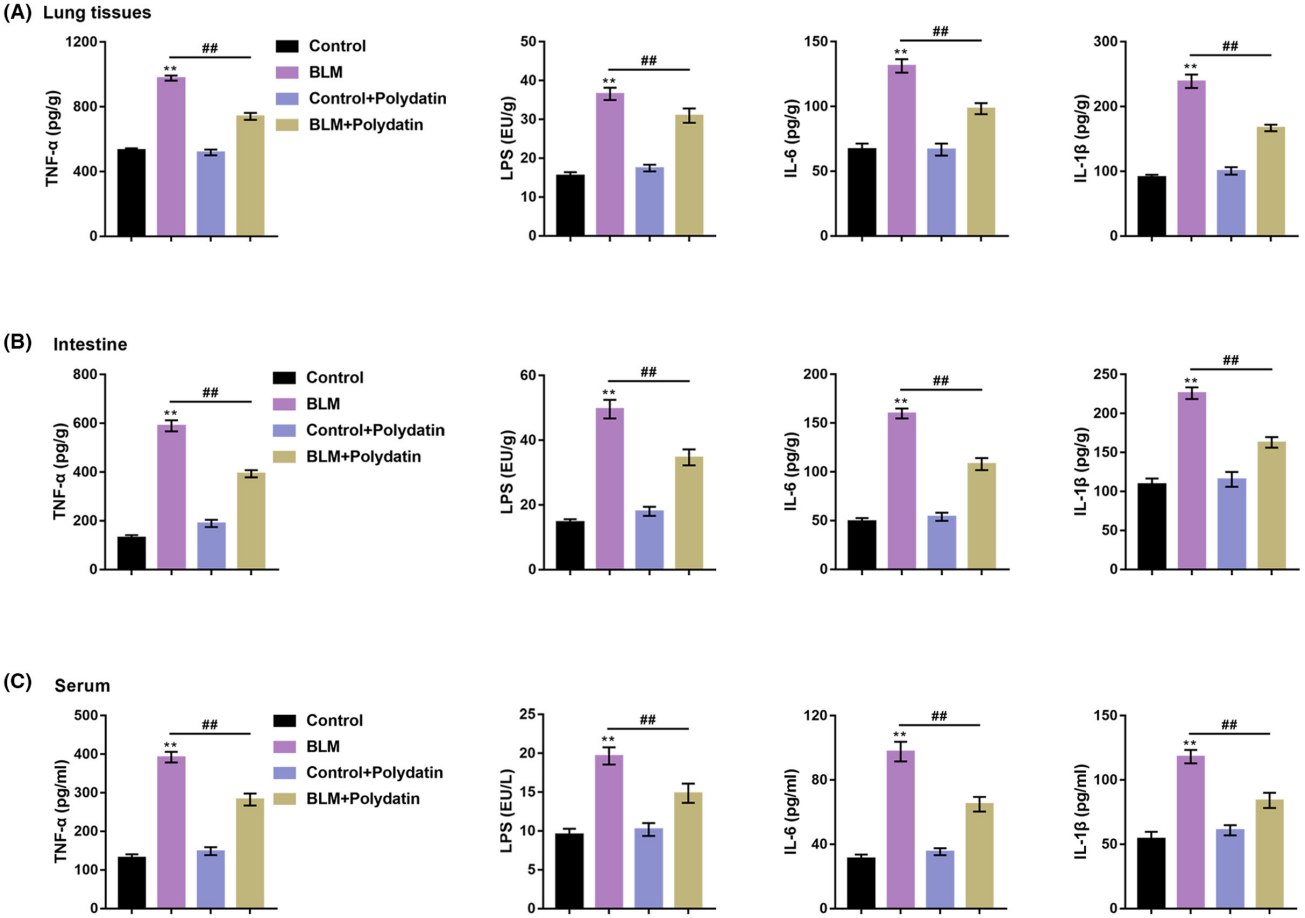
Effects of polydatin on inflammatory factors in the BLM model. The inflammatory factors TNF‐α, LPS, IL‐6, and IL‐1β in lung tissue (A), intestine (B), and serum (C) of mice in each group were detected by ELISA. **p < 0.01, compared with the control group; *##p <* 0.01, compared with the BLM group.

To further evaluate the pulmonary inflammation of the mice in each group, we used ELISA to detect the levels of TNF‐α, LPS, IL‐6 and IL‐1β in the lung tissue, intestine and serum of the mice. Figure 2A–C shows that compared with the control group, the contents of TNF‐α, LPS, IL‐6 and IL‐1β were significantly upregulated in the BLM group. However, compared with the BLM group, polydatin treatment significantly downregulated the levels of TNF‐α, LPS, IL‐6 and IL‐1β, indicating that polydatin could alleviate inflammation in BLM mouse model.

### 3.3 Quality control of 16S rRNA gene sequencing data

**FIGURE 3:**
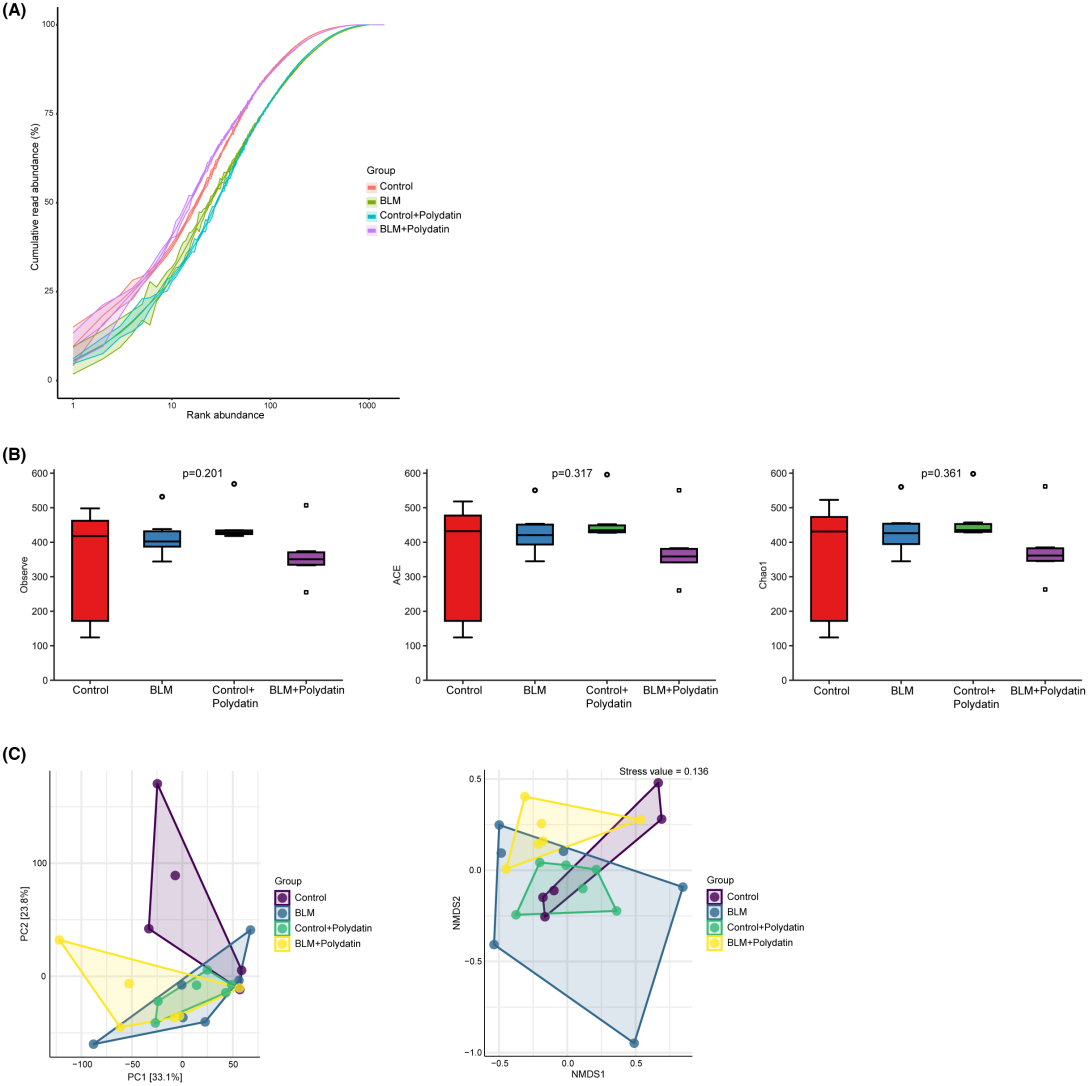
Quality control of 16S rRNA gene sequencing data. (A). Rarefaction curves (rank‐abundance) showing the richness (abundance) of species in the samples. (B). The α‐diversity analysis, including observed species, ACE, and Chao 1. (C). The β‐diversity analysis, including PCoA and NMDS analyses.

First, rarefaction curves (rank‐abundance) were used to assess whether the sequencing volume was sufficient to cover the species abundance in the sample. Figure 3A shows that the curves of the four groups of samples all tended to be flat, indicating that the sequencing depth covered enough species in the samples. Figure 3B shows the α‐diversity analysis results, and the observed species ACE and Chao1 were used to reflect the number of species in the community; no differences in the diversity of gut microbiota were observed in mice within the groups. The β‐diversity could reflect the differences in microbial composition among communities. In Figure 3C, the left panel represents the PCoA analysis, and the right panel represents the NMDS analysis. These results indicated intergroup differences in the gut microbial communities of mice among the different groups.

### 3.4 Dominant microflora species in different groups

**FIGURE 4:**
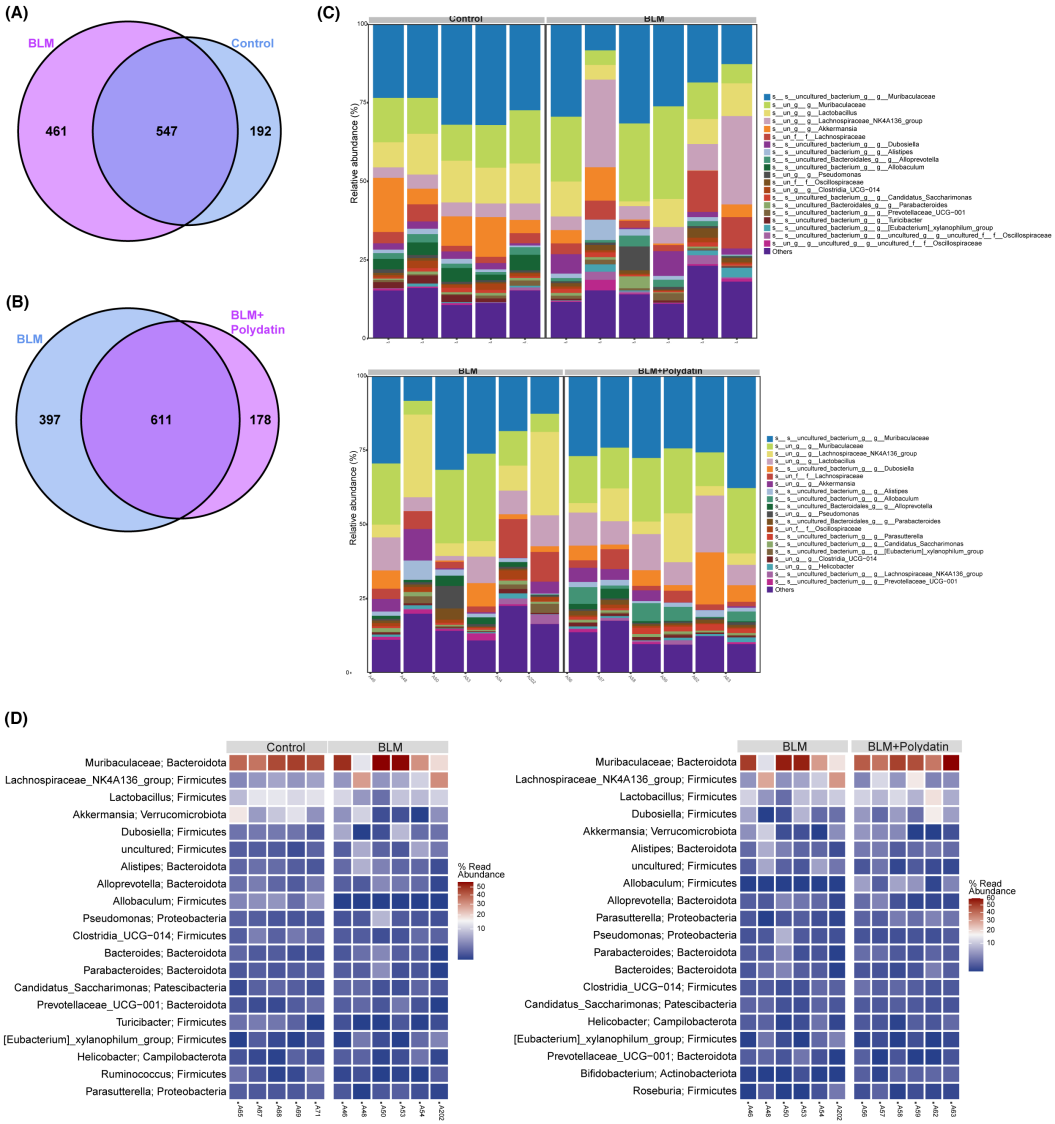
Dominant microflora species in different groups (A, B). Venn diagram shows the intersection of OTU abundances between the BLM group and the control group, the BLM group, and the BLM + PD group; (C, D). Top 20 species with the highest relative abundance between the BLM group and the control group, the BLM group and the BLM + PD group.

Due to the intergroup differences in the gut microbial communities of mice in each group, a Venn diagram was used to analyse shared microflora. As shown in Figure 4A,B, approximately 74% of the OTUs were consistent between the BLM and control groups. In comparison, approximately 77% of the OTUs were consistent between the BLM and BLM + PD groups. These results indicate that the BLM model and polydatin treatment significantly affected gut microbiota composition. Figure 4C,D depicted the top 20 species with the highest relative abundance. The dominant species in the control group were mainly *Muribaculaceae*, *Lactobacillus*, *Lachnospiraceae*, *Akkermansia* and *Allobaculum*. The main dominant species in the BLM model group were similar to those in the control group, and the abundance of *Pseudomonas*, *Xylanophilum* and *Parabacteroides* was relatively high in individual samples. The dominant species in the BLM + polydatin group were *Muribaculaceae, Lachnospiraceae*, *Lactobacillus*, *Dubosiella*, *Akkermansia*, *Alistipes*, *Allobaculum* and *Parasutterella*. Among these microflora, *Akkermansia*, *Lactobacillus* and *Dubosiella* are probiotics, and *Lachnospiraceae* is a pernicious bacterium. These results indicated that the BLM model and polydatin could alter the dominant microflora in the gut microbiota.

### 3.5 Specific effects of BLM and polydatin on the abundance of the dominant species of mouse gut microbiota

**FIGURE 5:**
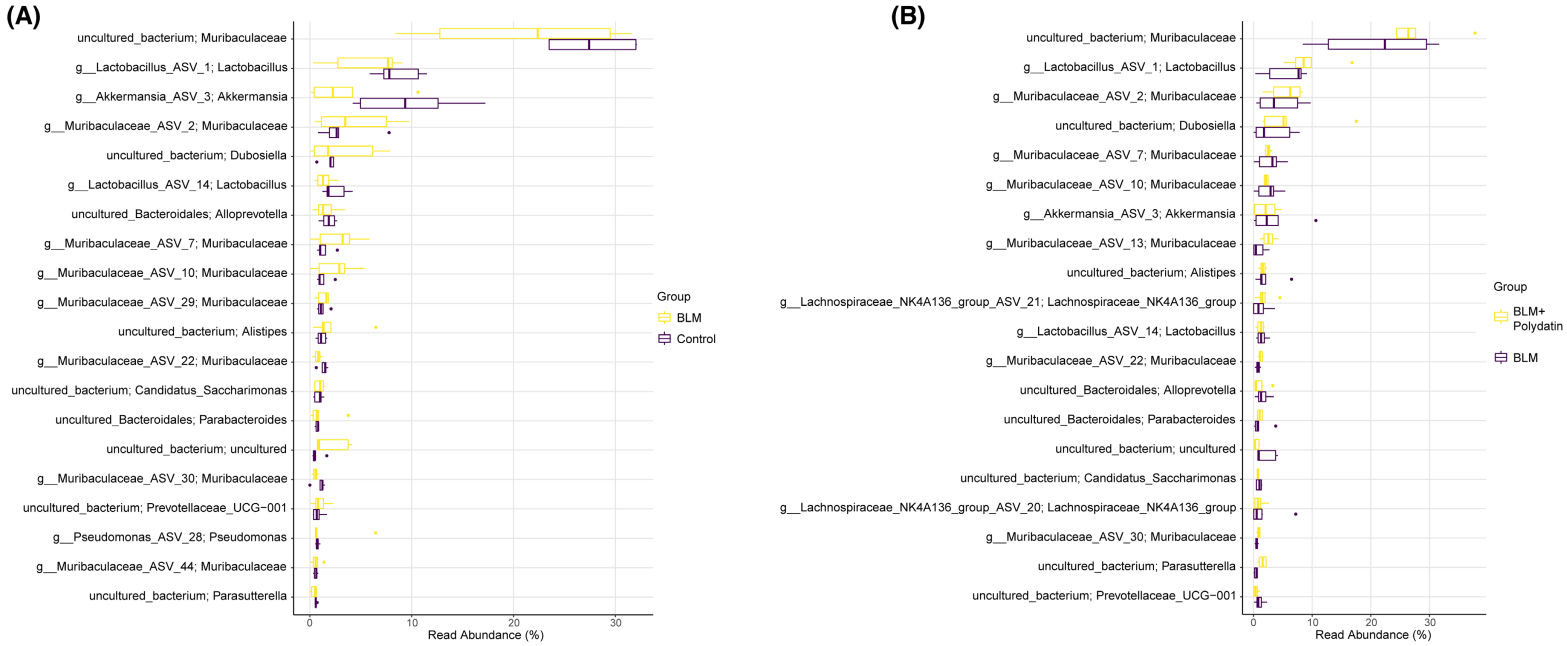
Specific effects of BLM and polydatin on the abundance of the dominant species of mouse gut microbiota. (A, B). Box plots show the abundance of the top 20 gut microbiota shared by different groups of mice.

The results mentioned above reflected that BLM and polydatin could affect the dominant species of the mouse gut microbiota. Next, the effects of BLM and polydatin on the abundance of dominant species were analysed. Figure 5A shows that compared with the control group, the probiotics *Muribaculaceae*, *Lactobacillus*, *Akkermansia*, *Alloprevotella* and other flora were less abundant in the BLM group. However, compared with the BLM group, the abundance of probiotics *Muribaculaceae*, *Lactobacillus*, *Akkermansia*, *Alloprevotella* and other flora in the BLM + polydatin group increased significantly (Figure 5B). These results suggest that BLM might affect the progression of PF and the abundance of gut probiotics and that polydatin could partially attenuate the effects of BLM on PF and gut probiotics.

### 3.6 Effects of polydatin‐treated mice's gut microbiota on PF

**FIGURE 6:**
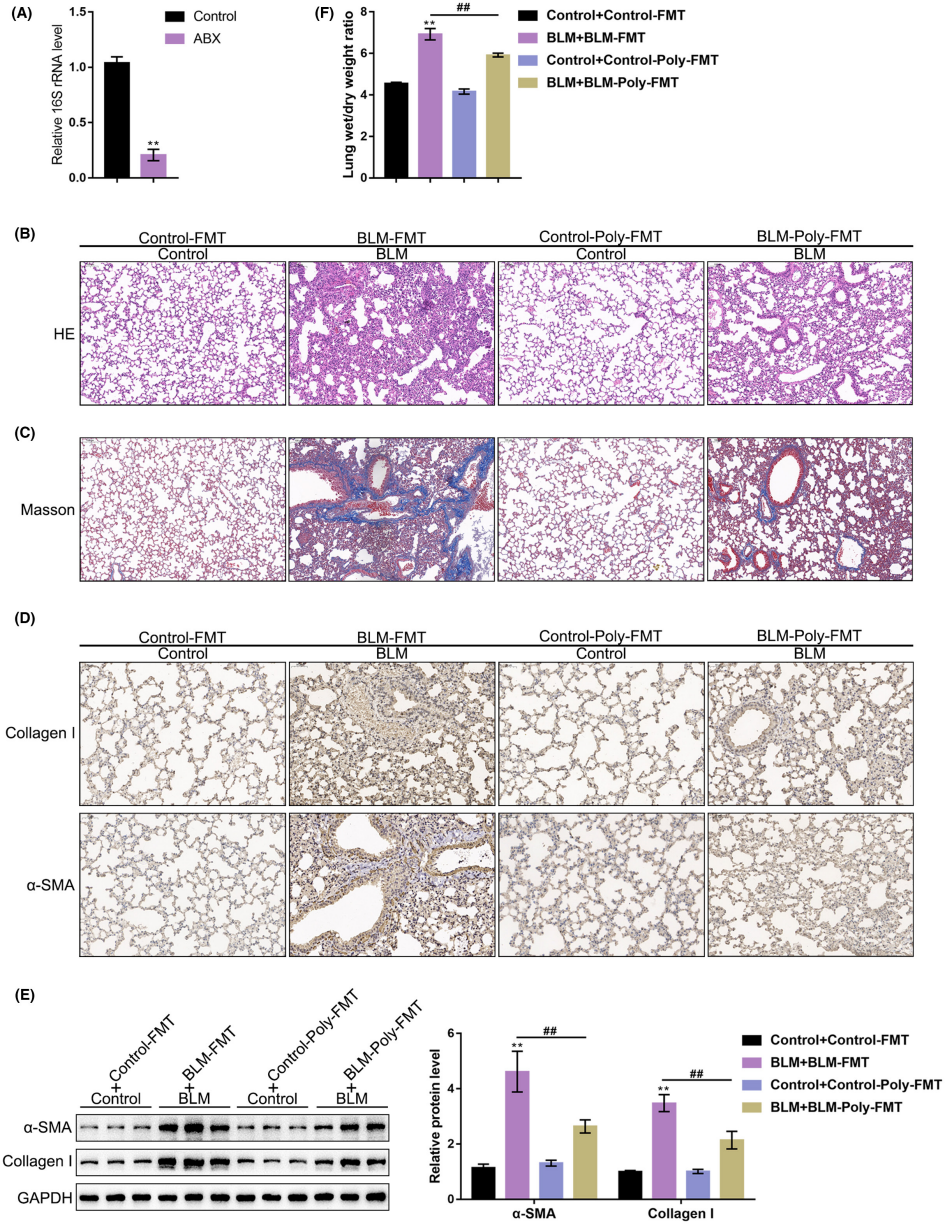
Effects of polydatin‐treated mice's gut microbiota on PF. (A). The expression of 16S rRNA in mouse faeces was measured by qRT–PCR, **p < 0.01, compared with the control group. (B). Haematoxylin and eosin staining was performed to detect the pathological changes in the lung tissue of the mice in each group. (C). Masson staining was performed to detect collagen deposition in the lung tissue of mice in each group. (D). IHC was performed to detect the expression levels of collagen I and α‐SMA in each group. (E). Western blotting was performed to examine the protein levels of collagen I and α‐SMA in each group. (F). Analysis of the W/D ratio of lung tissue in each group. **p < 0.01 versus Control + Control‐FMT group; ##*p* < 0.01 versus BLM + BLM‐FMT group.

The microflora extracted from the faeces of the control group, BLM group, control + polydatin group, and BLM + polydatin group were named control FMT, BLM‐FMT, control‐Poly‐FMT and BLM‐Poly‐FMT, respectively. These microflorae were transplanted into normal or BLM model mice treated with Abx. First, the depletion efficiency of ABx treatment was measured using qRT–PCR. As shown in Figure 6A, the 16S rRNA level in the Abx group was significantly reduced compared with that in the control group, indicating that the depletion of gut microbiota was successful and that mice were ready for FMT. The Abx‐treated mice were divided into four groups, and each group was named according to the control or BLM model and the origin of faecal microbiota to be transplanted: (1) Control + Control‐FMT: (2) BLM + BLM‐FMT: (3)Control + Control‐Poly‐FMT; and: (4) BLM + BLM‐Poly‐FMT. Figure 6B,C shows that Control‐Poly‐FMT caused no significant changes in control mice. The alveoli in the BLM + BLM‐FMT group were significantly thickened, and the deposition of fibroblasts and alveolar collagen was significantly increased compared with that in the Control + Control‐FMT group. Fibroblasts and alveolar collagen deposition were significantly reduced in the BLM + BLM‐poly‐FMT group compared with the BLM + BLM‐FMT group. Figure 6D,E shows consistent trends in which BLM‐Poly‐FMT significantly reduced the expression of collagen I and α‐SMA compared with the BLM + BLM‐FMT group. In addition, BLM‐Poly‐FMT significantly reduced the W/D ratio of the lung tissue compared with the BLM + BLM‐FMT group (Figure 6F). These results indicate that the gut microbiota derived from the faeces of mice in the BLM + polydatin group can effectively protect the lung tissue of mice from damage caused by BLM stimulation.

## 4 DISCUSSION

Polydatin has good therapeutic effects on several diseases, including severe traumatic brain injury‐induced neuronal injury and sepsis‐induced acute lung injury. Polydatin ameliorates pathological lung injury, promotes the recovery of pulmonary vascular permeability, reduces the oxidative stress response, decreases the release of inflammatory cytokines (IL‐6, IL‐1, TNF‐α and MCP‐1) and suppresses the formation of reticular structures in lung tissue.^19^ Polydatin also attenuates PM2.5‐induced lung injury and downregulates leukocytes, inflammation‐related lipids and lung proinflammatory cytokines in bronchoalveolar lavage fluid (BALF).^20^ Regarding radiation‐induced lung injury, polydatin attenuates radiation‐induced lung injury by inhibiting epithelial‐mesenchymal transition (EMT) and increasing Sirt3 expression.^21^ For PF, chronic inflammation is a necessary component.^22^ Researchers found that polydatin significantly reduced the levels of hydroxyproline, TNF‐α, IL‐6, IL‐13, myeloperoxidase, and malondialdehyde and promoted the activity of total superoxide dismutase in the lung tissue. Moreover, polydatin reduced the lung damage caused by BLM‐induced PF by inhibiting TGF‐β1 expression and the phosphorylation of Smad 2/3 and ERK‐1/2 in vivo.^23^ Consistently, this study found that polydatin significantly attenuated the lung injury induced by BLM and reduced the amount of TNF‐α, LPS, IL‐6 and IL‐1β in the lung, intestine and serum. These findings confirmed the therapeutic effects of polydatin on BLM‐induced PF; however, the underlying mechanism remains unclear.

The gut microbiota is acknowledged as a key innate immune system regulator that is engaged in the immunological development of the intestinal mucosa and impacts immune system development.^17^, ^24^ In addition, the microbiota that has colonized the mucosa of the respiratory and digestive systems can regulate tissues and constitute the material foundation for lung‐gut linkages. For example, gavage with a suspension of faeces from healthy mice can reduce the symptoms of pneumonia in mice infected with *Streptococcus pneumoniae* who are receiving antibiotic therapy.^25^, ^26^ Oral *Lactobacillus* and *Bifidobacterium* supplementation in children can help improve asthma symptoms and reduce seizure frequency.^27^ Regarding the relationship between IPF and gut microbiota, it has been recently suggested that the microbiome could actually influence the risk of initiation and/or progression of IPF,^28^, ^29^, ^30^, ^31^ where gut and airway microbiota are considered relevant players. In this study, we collected the faeces of a mouse model in different groups and performed 16S rDNA sequencing and untargeted metabolomics to investigate the effects of BLM on the mouse gut microbiota.

BLM stimulation with or without polydatin treatment caused no significant changes in gut microbiota diversity; however, the dominant species and corresponding abundance were altered. Although BLM mice with or without polydatin treatment shared the most dominant species, *Dubosiella*, a type of probiotic, only existed in the polydatin treatment group. *Dubosiella*, belonging to the family *Erysipelotrichaceae*, was found to be significantly reduced in high‐fat diet‐ and STZ‐induced diabetic mice.^32^ A previous study indicated that vitamin K2 supplementation could increase the abundance of intestinal *Dubosiella* and decrease the relative expression of activating transcription factor 4 to inhibit blood pressure in mice.^33^ Interestingly, other probiotics, such as *Muribaculaceae*, *Lactobacillus*, *Akkermansia* and *Alloprevotella*, were detected in all BLM mice but with altered abundance in the presence or absence of polydatin treatment. Polydatin treatment significantly increased the abundance of *Muribaculaceae*, *Lactobacillus*, *Akkermansia*, and *Alloprevotella* compared with the BLM group, indicating that polydatin could affect the composition of the mouse gut microbiota by increasing probiotic abundance. *Lactobacillus* was shown to protect mice against influenza virus‐induced pathology and mortality.^34^ *Muribaculaceae* mediated salmon peptide fraction‐induced improvement of metabolic syndrome.^35^ Faecal *Akkermansia muciniphila* was associated with the clinical benefit of immune checkpoint inhibitors in patients with non‐small cell lung cancer or kidney cancer.^36^ The abundance of *Alloprevotella* was reported to be decreased in COPD patients with declining function.^37^ Considering these findings, polydatin might improve BLM‐induced PF by affecting the mouse gut microbiota.

Complementary probiotics or FMT have been proven to restore the gut microecology and reduce inflammation in acute lung injury and pneumococcal pneumonia.^17^, ^38^, ^39^ Some evidence suggests a relationship between diet and PF, and dietary factors could regulate the gut‐lung axis, which in turn affects PF development.^40^ To address the speculation that polydatin might improve BLM‐induced PF by affecting the mouse gut microbiota, we performed FMT using faeces obtained from mice in different groups and observed beneficial effects on BLM mice caused by BLM‐Poly‐FMT. Similar to polydatin treatment, BLM‐Poly‐FMT improved lung tissue histopathological features, decreased the W/D ratio, and reduced the levels of collagen I and α‐SMA. These findings indicate that BLM‐Poly‐FMT, obtained from the polydatin‐treated BLM mouse model, ameliorated BLM‐induced lung injury in PF model mice.

In conclusion, polydatin alleviated BLM‐induced PF and altered gut microbiota species abundance. Moreover, polydatin‐induced alteration of the gut microbiota may also be involved in the therapeutic effect of polydatin in PF.

## AUTHOR CONTRIBUTIONS

**Jia Yang:** Conceptualization (equal); formal analysis (equal); methodology (equal); writing – original draft (equal). **Xiawei Shi:** Data curation (equal); methodology (equal); validation (equal); visualization (equal). **Rundi Gao:** Data curation (equal); methodology (equal). **Liming Fan:** Investigation (equal); methodology (equal). **Ruilin Chen:** Investigation (equal); visualization (equal). **Yu Cao:** Investigation (equal). **Tingzhen Xu:** Conceptualization (equal); writing – review and editing (equal). **Junchao Yang:** Conceptualization (equal); funding acquisition (equal); supervision (equal); writing – review and editing (equal).

## CONFLICT OF INTEREST STATEMENT

The authors declare no conflicts of interest in this work.

## Data Availability

The data that support the findings of this study are available from the corresponding author upon reasonable request.
